# Differential effects of COVID-19 lockdowns on well-being: interaction between age, gender and chronotype

**DOI:** 10.1098/rsif.2021.0078

**Published:** 2021-06-02

**Authors:** Shay Oved, Merav Mofaz, Anat Lan, Haim Einat, Noga Kronfeld-Schor, Dan Yamin, Erez Shmueli

**Affiliations:** ^1^ Department of Industrial Engineering, Tel-Aviv University, Tel-Aviv, Israel; ^2^ School of Zoology and Sagol School of Neuroscience, Tel-Aviv University, Tel-Aviv, Israel; ^3^ Center for Combating Pandemics, Tel-Aviv University, Tel-Aviv, Israel; ^4^ School of Behavioral Sciences, The Academic College of Tel Aviv-Yafo, Tel-Aviv, Israel

**Keywords:** COVID-19, lockdown, smartwatch, differential effects

## Abstract

The unprecedented restrictions imposed due to the COVID-19 pandemic altered our daily habits and severely affected our well-being and physiology. The effect of these changes is yet to be fully understood. Here, we analysed highly detailed data on 169 participants for two to six months, before and during the second COVID-19 lockdown in Israel. We extracted 12 well-being indicators from sensory data of smartwatches and from self-reported questionnaires, filled daily using a designated mobile application. We found that, in general, lockdowns resulted in significant changes in mood, sleep duration, sport duration, social encounters, resting heart rate and number of steps. Examining subpopulations, we found that younger participants (aged 20–40 years) suffered from a greater decline in mood and number of steps than older participants (aged 60–80 years). Likewise, women suffered from a higher increase in stress and reduction in social encounters than men. Younger early chronotypes did not increase their sleep duration and exhibited the highest drop in mood. Our findings underscore that while lockdowns severely impacted our well-being and physiology in general, greater damage has been identified in certain subpopulations. Accordingly, special attention should be given to younger people, who are usually not in the focus of social support, and to women.

## 1. Introduction

Restrictions against the COVID-19 pandemic have altered everyday life in many ways. Our daily habits—the time we go to sleep and wake up, eat, commute, work and engage in social activities—all changed as part of the battle against the disease, with many countries imposing social distancing regulations and mobility restrictions such as lockdowns [1–3]. These changes are expected to affect our well-being and physiology. Importantly, in the context of contracting and fighting viral infections, these changes can also weaken our immune system. Thus, it is imperative to understand the complex consequences of social distancing and lockdowns.

Recent studies exploring the influence of COVID-19 on well-being found both negative and positive effects. Some studies found that lockdowns were associated with adverse outcomes such as high levels of anxiety and stress, and lower physical activity [4–6]. By contrast, lockdowns were also correlated with favourable changes in sleep patterns, including longer sleep duration [7–10]. Moreover, some of the documented effects of lockdown are inconsistent. For example, Leone found a positive, albeit small, impact of lockdowns on sleep quality [10], whereas others reported an adverse opposite change [5,8,11], or no change at all [12]. Such discrepancies could be explained by variability in the response of subpopulations.

Better understanding of the different effects of social distancing and lockdowns and their consequences on diverse populations can have a critical impact on the recommended behaviour at the individual level, with the possibility of specific directions, instructions, medical advice and social care to separate sub-groups. Furthermore, it may also bare important insights for our ‘new normal’, post-COVID-19 life. Only a few studies thus far examined the differential effects of the social changes induced by the disease between subpopulations. Several studies suggested more profound effects in younger adults. Specifically, lockdowns were associated with higher depressive symptoms, stress and anxiety, lower sleep quality levels, longer sleep duration and later sleep onset and offset in younger compared to older participants [10,12–14]. Several other studies reported women are at greater risk for negative effects of lockdowns on mental health [13,14]. It is important to note that all these studies were conducted during the first lockdown, comparing pre-COVID-19 life to lockdowns. Our study was conducted after the first lockdown, during the COVID-19 pandemic, and therefore is the first to decipher the specific effects of the lockdown from the general effects of the pandemic, and focuses on the long-lasting effects of lockdowns rather than on the acute, life-threatening feeling people had on the first lockdown. Such knowledge is highly important for decision makers, social and health services, as well as the public.

The individual’s chronotype is another important variable that may moderate the effects of lockdown. Human sleep–wake behaviour is timed by the interaction between our intrinsic biological clock synchronized to the light/dark cycle, a homeostatic sleep drive, and social schedules [15,16]. The innate timing of sleep can be described by an individual’s chronotype and can be classified as ‘early’ or ‘late’ relative to people measured under similar conditions [17]. Early chronotypes spontaneously wake up early in the morning and go to sleep early at night, while late chronotypes wake up late in the morning (or noon) and go to sleep late at night or early morning. Differences between chronotypes were found in the timing of many daily rhythms peak times (acrophase). For instance, the acrophase of body temperature, melatonin and cortisol levels and even cognitive function occur earlier in early chronotypes compared with late chronotypes [18–20]. The distribution of chronotypes within human population is almost normal with a slight over-representation of later chronotypes. The distribution is derived from genetic polymorphisms in clock genes [21,22], age [23] and light exposure [23]. Because most of us live according to the same social time, late chronotypes often experience a greater misalignment between their internal biological time and external work/social time. Such misalignment is termed ‘social jetlag’ (SJL) [24]. A growing body of literature has found associations between a later chronotype and increased risk for depression and mood disorders, higher metabolic dysfunction rates and morbidity, and reduced physical activity [17,25–27]. COVID-19 lockdowns have changed both our social schedules and the synchronizers of our biological clock, including light exposure: most of us spend less time outdoors exposed to sunlight during the day, but are exposed to artificial light during the night. Such changes are expected to affect different chronotypes differently. However, only two studies [10,12] thus far studied the relationship between chronotypes and lockdowns, focusing on the effects of lockdowns on sleep timing. These studies, which used self-reported questionnaires, found a profound delay in sleep onset time in response to lockdowns in all chronotypes.

The COVID-19 pandemic provides a unique opportunity to test the effects of social and mobility changes on well-being. Importantly, these effects may also indirectly affect the immune system: sleep, biological rhythms and well-being are all interconnected, and affect our immune system function and ability to fight viral infections [28–34]. Therefore, understanding these relations may also help us develop protective recommendations for reducing the risk of infection and improve vaccination outcomes [33], which may be different for different subpopulations.

We specifically sought to probe whether age, gender and chronotype moderate the effects of such changes on well-being using both wearable monitoring devices (Fitbit) and daily self-reported digital questionnaires. Data were collected from 169 participants between 11 May to 17 October 2020, before and during the second COVID-19 lockdown in Israel, which was imposed on 18 September 2020. During the lockdown period, restaurants, schools, hotels, fitness clubs, swimming pools, malls and all other facilities (except for food stores and pharmacies) were closed, and people’s movement was restricted to 500 m from their homes (except for commuting to work and to other essential activities). We used the collected data to evaluate the change in various well-being indicators, including sleep patterns, physical activity and mood, during the lockdown period.

## 2. Material and methods

### 2.1. Study design

In the current study, we analyse data that were collected as part of the PerMed pilot study [35], to test the effects of the second COVID-19 lockdown in Israel on various indicators of well-being in different subpopulations. The study includes 192 participants above the age of 18 who joined the study for two to six months and were equipped with our dedicated mobile application—The PerMed App, and a Fitbit^®^ Inspire HR smartwatch. The study was approved by Tel-Aviv University Institutional Review Board (IRB) and was conducted under strict protocol guidelines.

In order to recruit subjects and keep them engaged throughout the study, we hired a professional survey company. The survey company used advertisements on social media for recruitment of people from the general population. The survey company was responsible to guarantee that participants met the study’s requirements, including their willingness to fill a daily questionnaire and wear a smartwatch during the entire study. No additional filtering of participants was performed. Eligible participants were met face-to-face and received a detailed explanation about the study, after which they were requested to sign a consent form. Then, participants were asked to fill a one-time enrolment questionnaire and to install two apps on their smartphones: the Fitbit app which was used to collect data from their smartwatch, and the dedicated PerMed app which we developed to allow participants to fill the daily questionnaires.

In order to improve the quality and reliability of the data and to ensure its continuous collection, we applied the following measures. First, participants who did not fill the daily questionnaire by 19.00, received a notification in their mobile app to fill the questionnaire. Second, we developed a dedicated dashboard. The dashboard, which was monitored regularly, helped us identify data collection issues, such as participants who did not fill the daily questionnaires or participants who did not wear their smartwatches. Such participants were contacted by the survey company, and were encouraged to cooperate better. The dashboard also helped us to identify issues that were not related to participants’ cooperation, such as bugs in the mobile app. This identification allowed us to respond faster and provide timely solutions.

As for the time periods of the study, we considered the following: (1) the period from the beginning of the PerMed pilot study—11 May 2020, and a week before the beginning of the second COVID-19 lockdown in Israel—11 September 2020, and (2) the lockdown period—18 September 2020 to 17 October 2020. During the week between 12 September 2020 and 18 September 2020, the intention to conduct a national lockdown has been widely discussed in the media. Thus, to neutralize the announcement and discussion’s effects, we did not analyse data from this specific week.

### 2.2. Data collection

In this study, we analysed two sources of data that were collected as part of the PerMed pilot study:

— The daily questionnaire consisted of eight questions about mood, stress, sport duration, sleep duration, sleep quality, encounters with other people, clinical symptoms and diagnosed diseases. A detailed description of the questionnaire is provided in electronic supplementary material, A.
— Smartwatch data were collected at a daily aggregation level, and consisted of average resting heart rate, active minutes, steps, distance, calories and sleep data, including bed times, wake times, time in bed and total sleep duration.

### 2.3. Data preprocessing

Before analysing the data, we performed several preprocessing steps. First, in cases where participants filled the daily questionnaire more than once on the same day, only the latest answers for that day were considered. The rationale behind this decision was that a questionnaire, once filled, was sent to the server, and could not be updated anymore. Therefore, in case of a filling error, participants were instructed to re-fill the questionnaire.

Next, sleep records were processed to identify the main sleep intervals. More specifically, in the vast majority of cases participants had at most one sleep record per day. However, in roughly 20% of the cases, sleep data collected by the smartwatch included more than one sleep record for the same participant for the same day. This could be due to participants waking up once or more during their main sleep, or if they had afternoon naps in addition to their main sleep. To identify the segments belonging to the main sleep, we first merged consecutive sleep records with a difference of up to 2 h between them into a single sleep interval. Then, sleep intervals with a total duration of fewer than 3 h were omitted. We also excluded from the analyses sleep records with start time between 10.00 and 16.00, assuming they represent nap times. This last step resulted in omitting 0.17% of the participant-day sleep records.

Then, for each participant, we calculated nine well-being indicators. The first six indicators come from the daily self-reported questionnaire. They include: mood (on a scale of −2 to 2, where −2 means awful and 2 means excellent), stress (on a scale of −2 to 2, where −2 means very low and 2 means very high), sleep quality (on a scale of −2 to 2, where −2 means awful and 2 means excellent), sleep duration (hours), sport duration (minutes) and the number of social encounters. The other three indicators were extracted from the smartwatch, and include: resting heart rate (bpm), steps and sleep duration (hours). We calculated the mean value for each participant and each of the nine indicators, before and during the lockdown.

In order to capture changes in sleep routines, we also calculated the following three indicators, based on smartwatch data:

1. Mid sleep point free days (MSF)—For each participant and for each week, we calculated the weekly MSF value as the average of middle sleep time in free days (weekends and public holidays). The middle sleep time for a given day was calculated as the halfway point between sleep onset and sleep offset. Then, the MSF value for that participant was calculated as the average over all weekly MSF values—once for the period before the lockdown and once for the period during the lockdown. For the calculation, Jewish weekends (Friday and Saturday) and holidays were marked as free days.
2. Mid sleep point work days (MSW)—For each participant and for each week, we calculated the weekly MSW value as the average of middle sleep time in work days. Then, the MSW value for that participant was calculated as the average over all weekly MSW values—once for the period before the lockdown and once for the period during the lockdown. The calculation of MSW for a given week was done only if the participant had at least two sleep records in this week’s workdays.
3. SJL—For each participant and for each week that had a weekly MSF value and a weekly MSW value, we calculated the weekly SJL value as the absolute difference between them. Then, the SJL value for that participant was calculated as the average over all weekly SJL values—once for the period before the lockdown and once for the period during the lockdown.

Finally, 23 of 192 participants were omitted from the analysis because they lacked MSF information in the period before lockdown (most probably because they barely wore their smartwatch at nights or weekends).

### 2.4. Statistical analyses

In order to test the effect of the lockdown on different subpopulations for different well-being indicators, we applied a mixed ANOVA test. More specifically, we considered the 12 well-being indicators mentioned in the previous subsection. For each of these 12 indicators, we used a separate mixed ANOVA test, where the dependent variable was the indicator, and the four independent variables (main factors) were:

1. Lockdown—a within-subjects factor with two levels: before lockdown and during lockdown.
2. Age group—a between-subjects factor with two levels: younger and older. The groups were divided based on the median value.
3. Gender—a between-subjects factor with two levels: men and women.
4. Chronotype—a between-subjects factor with two levels: early chronotype and late chronotype. The groups were divided based on the median value of MSF before the lockdown.^1^

More formally, for each of the 12 well-being indicators, the considered mixed ANOVA model includes the four main factors and all of their potential interactions:

2.1 indicator∼lockdown ∗ age_group ∗ gender ∗ chronotype

We performed *post hoc* multiple comparison Bonferroni tests for the significant interactions, and measured Cohen’s *d* effect size for the significant effects. Statistical analyses were performed using IBM SPSS Statistics v. 25.

## 3. Results

### 3.1. Descriptive statistics

**Figure 1.**
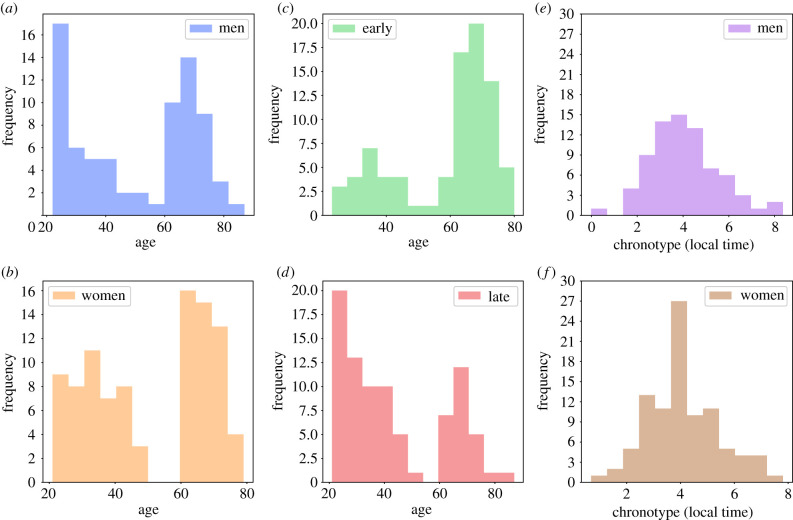
Distribution of subpopulations: (*a*)+(*b*) distribution of age divided by gender, (*c*)+(*d*) distribution of age divided by chronotype, and (*e*)+(*f*) distribution of chronotype divided by gender.

Out of the 169 participants, 94 (55.62%) were women, and 75 (44.38%) were men. The age of the participants ranged between 20 and 80, with clear two main age groups: 20–40 and 60–80. The chronotypes of participants were characterized by a Gaussian shape, with a mean of 4.05 local time (figure 1). More details about the size of each subpopulation are provided in electronic supplementary material, B.

**Figure 2.**
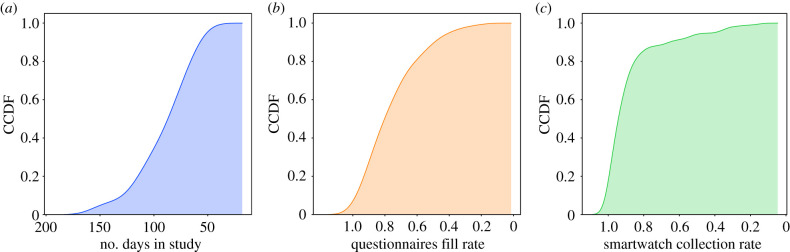
Data quality: (*a*) days in study, (*b*) questionnaires fill rate, (*c*) smartwatch collection rate. The *y*-axis represents the complementary cumulative distribution function (CCDF)—i.e. the ratio of participants with the corresponding value on the *x*-axis or above.

As can be seen from figure 1: (1) the two age groups distribute roughly evenly between men and women^2^ (figure 1*a*,*b*); (2) the younger age groups is mainly associated with late chronotypes while the older age group is mainly associated with early chronotypes (figure 1*c*,*d*); and (3) women and men present a similar distribution of chronotypes (figure 1*e*,*f*).

Unsurprisingly, the two age groups also differ in the number of young children they have, where the younger age group is characterized by a significantly higher number of young children. For example, the average number of children under the age of 4 is 0.30 for the younger age group and 0.03 for the older age group (unequal variances *t*-test, *p*-value < 0.01). Similarly, the average number of children between the ages 5–17 is 0.92 for the younger age group and 0.06 for the older age group (unequal variances *t*-test, *p*-value < 0.01).

Figure 2 illustrates the quality of the collected data. Figure 2*a* shows the distribution of the number of days each participant spent in the study. Participants spent on average 91.21 ± 27.12 days in the study, where the vast majority of them spent at least two months. Figure 2*b* presents the distribution of the daily questionnaire’s fill rate. As can be seen from the figure, the vast majority of participants filled the daily questionnaires in more than 60% of the days they spent in the study. Figure 2*c* presents the distribution of coverage rate for smartwatch data. As can be seen from the figure, the vast majority of the participants had smartwatch data for more than 80% of the days they spent in the study.

Table 1 presents information about the mean and 95% confidence interval for each of the 12 examined well-being indicators, for the time periods before and during the lockdown, for the entire population of 169 participants. As can be seen from the table, some indicators (sleep duration, MSF and MSW) seem to increase during the lockdown, while some other indicators (mood, encounters, resting heart rate, steps) seem to decrease. A breakdown of this table according to age group, gender and chronotype is provided in electronic supplementary material, B.

**Table 1.** Descriptive statistics. Each row represents a single well-being indicator and contains: the mean value before lockdown, the mean value during lockdown, the mean difference and the 95% confidence interval (lower bound and upper bound) of the difference.

	indicator	before lockdown	during lockdown	difference	CI LB	CI UB
questionnaire	mood	0.86	0.78	−0.08	−0.13	−0.02
	stress	−0.69	−0.69	0.00	−0.05	0.06
	sleep quality	0.47	0.49	0.02	−0.03	0.07
	sleep duration (hours)	6.29	6.42	0.13	0.05	0.20
	sport duration (minutes)	31.34	29.04	−2.31	−4.64	0.03
	encounters	12.09	7.86	−4.23	−5.36	−3.10
smartwatch	resting heart rate (bpm)	62.90	62.23	−0.67	−1.11	−0.23
	steps	8671.11	7967.67	−703.44	−1032.90	−373.98
	sleep duration (hours)	7.01	7.15	0.14	0.05	0.22
	MSF (local time)	4:05	4:16	0.19	0.07	0.31
	MSW (local time)	3:41	3:48	0.12	0.00	0.23
	SJL (hours)	0.84	0.86	0.02	−0.09	0.13

### 3.2. Within-subjects effects

Table 2 presents the *p*-values of the within-subjects effects for the mixed ANOVA test that was conducted for each of the 12 examined well-being indicators. Each row represents a single indicator (and thus also a single test) and each column represents the examined factor/interaction. Each entry represents the *p*-value of the corresponding effect, where statistically significant effects are marked with asterisks. A complementary table of the between-subjects effects is provided in electronic supplementary material, C.

**Table 2.** Within-subjects effects. Each row represents a mixed ANOVA test for a single well-being indicator. Each column represents a single factor/interaction, and each entry represents the p-value of the corresponding effect. Statistically significant effects are marked with asterisks. The direction of the effect for the statistically significant results are marked with arrows. In the case of interactions, Y indicates the younger age group, O indicates the older age group, M indicates men, W indicates women, E indicates early chronotypes and L indicates late chronotype.

	indicator	lockdown	lockdown*age group	lockdown*gender	lockdown*chronotype	lockdown*age group*gender	lockdown*age group*chronotype	lockdown*gender**chronotype	lockdown*age group*gender*chronotype
questionnaire	mood	0.00*** (↓)	0.05** (Y↓)	0.58	0.58	0.14	0.02** (Y:E↓)	0.80	0.77
	stress	0.90	0.36	0.01*** (M↓ W↑)	0.30	0.24	0.92	0.73	0.67
	sleep quality	0.69	0.47	0.11	0.16	0.14	0.21	0.16	0.40
	sleep duration	0.00*** (↑)	0.99	0.42	0.70	0.41	0.71	0.30	0.94
	sport duration	0.08* (↓)	0.14	0.45	0.52	0.71	0.89	0.32	0.14
	encounters	0.00*** (↓)	0.55	0.03** (M↓ W↓)	0.15	0.43	0.10	0.76	0.46
smartwatch	resting heart rate	0.05* (↓)	0.12	0.06* (W↓)	0.61	0.23	0.11	0.49	0.63
	steps	0.00*** (↓)	0.01* (Y↓)	0.74	0.41	0.30	0.76	0.86	0.11
	sleep duration	0.05** (↑)	0.60	0.57	0.35	0.32	0.04** (Y:L↑;O:E↑)	0.59	0.75
	MSF	0.02** (↑)	0.11	0.89	0.95	0.23	0.22	0.49	0.45
	MSW	0.07* (↑)	0.67	0.46	0.28	0.74	0.82	0.70	0.44
	SJL	0.76	0.19	0.80	0.59	0.69	0.16	0.74	0.46

*** *p* < 0.01, ** *p* < 0.05, * *p* < 0.1.

#### 3.2.1. Effects of the lockdown on the entire population

Overall, we found that the lockdown affected various well-being indicators in the entire population (table 2). We found a decline in mood (an average level of 0.87 versus 0.76, *p* < 0.01, Cohen’s *d* = 0.14), sport duration (an average number of 30.05 minutes versus 27.71 min, *p* = 0.08, Cohen’s *d* = 0.10), encounters (an average number of 11.49 encounters versus 7.81 encounters, *p* < 0.01, Cohen’s *d* = 0.44), resting heart rate (an average of 62.59 bpm versus 62.11 bpm, *p* = 0.05, Cohen’s *d* = 0.09), and steps (an average number of 8453.30 steps versus 7710.58 steps, *p* < 0.01, Cohen’s *d* = 0.19). An increase was observed in sleep duration based on questionnaire responses (an average of 6.28 h versus 6.42 h, *p* < 0.01, Cohen’s *d* = 0.14) and based on smartwatch records (an average of 7.03 h versus 7.12 h, *p* = 0.05, Cohen’s *d* = 0.14), MSF (an average hour of 4.03 versus 4.12, *p* = 0.02, Cohen’s *d* = 0.13) and MSW (an average hour of 3.43 versus 3.49, *p* = 0.07, Cohen’s *d* = 0.08). No significant effects were found for stress, sleep quality and SJL.

#### 3.2.2. Unequal effects of the lockdown on different subpopulations

Interestingly, beyond the general effects, the data clearly demonstrate dissimilar effects in different subpopulations. Moreover, when analysing separately the different subpopulations, the effect sizes (Cohen’s *d*) of the differences before and after lockdown become larger. Here, we focus on three different segmentations of the population—age group, gender and chronotype.

**Figure 3.**
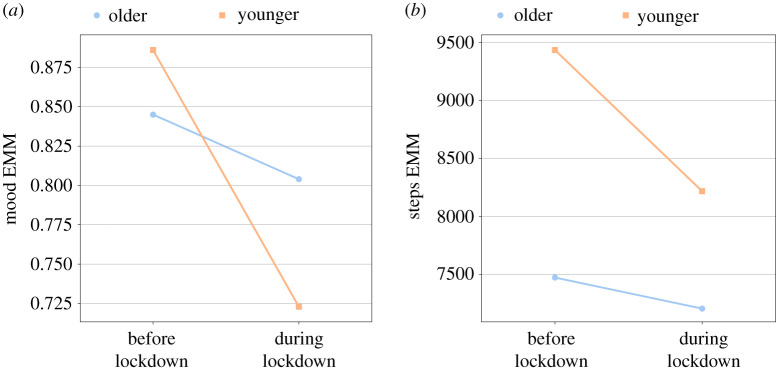
Effects of the lockdown on different age groups for: (*a*) mood and (*b*) steps. The *y*-axis represents the estimated marginal means (EMM) for the examined well-being indicator.

A significant interaction was found between lockdown and age group in mood (*p* = 0.05) and steps (*p* = 0.01), suggesting a different effect of the lockdown on the two age groups with respect to these two indicators. Specifically, we observe that the negative effect of the lockdown was stronger for the younger age group in both cases (figure 3*a*,*b*). *Post hoc* analysis revealed that the decline in mood and steps between the two time periods was significant in the younger age group (*p* < 0.01, Cohen’s *d* = 0.24 (mood); *p* < 0.01, Cohen’s *d* = 0.35 (steps)) but not in the older age group.

**Figure 4.**
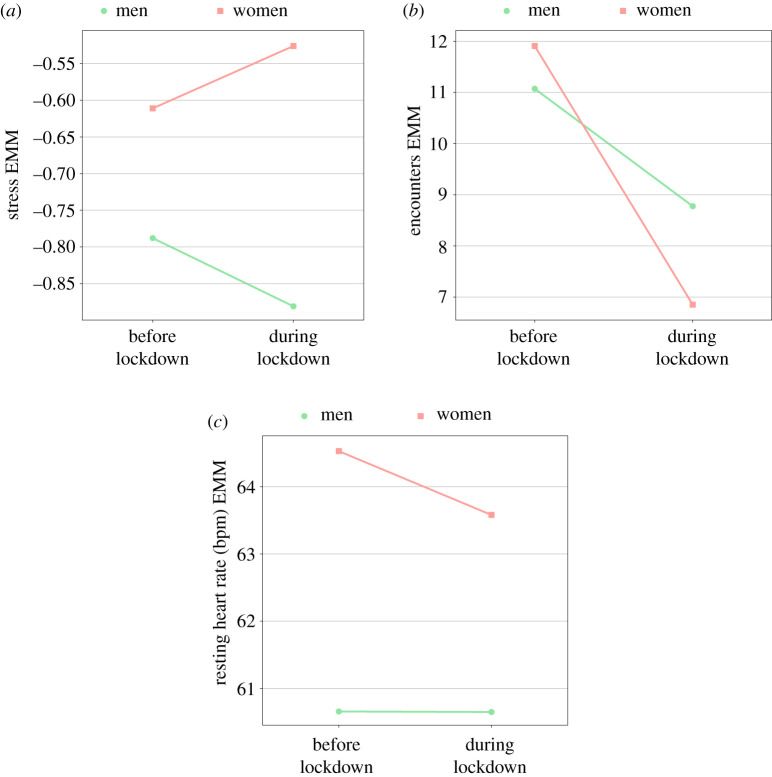
Effects of the lockdown on different genders for: (*a*) stress, (*b*) encounters and (*c*) resting heart rate. The *y*-axis represents the estimated marginal means (EMM) for the examined well-being indicator.

A significant interaction was found between lockdown and gender in stress (*p* = 0.01), encounters (*p* = 0.03) and resting heart rate (*p* = 0.06), suggesting a different effect of the lockdown on women and men with respect to these three indicators. Specifically, we observe that men became less stressed during the lockdown while women became more stressed (figure 4*a*); the number of encounters declined for both genders, but for women the decline was sharper (figure 4*b*); and women’s heart rate declined during lockdown while men’s heart rate did not change (figure 4*c*). *Post hoc* analysis revealed that the change of stress between the two time periods was significant for women (*p* = 0.04, Cohen’s *d* = 0.14) and men (*p* = 0.06, Cohen’s *d* = 0.17), but the change was in different directions. The change in encounters was also significant for women (*p* < 0.01, Cohen’s *d* = 0.56) and men (*p* = 0.02, Cohen’s *d* = 0.30). The change in resting heart rate was significant for women only (*p* < 0.01, Cohen’s *d* = 0.14).

**Figure 5.**
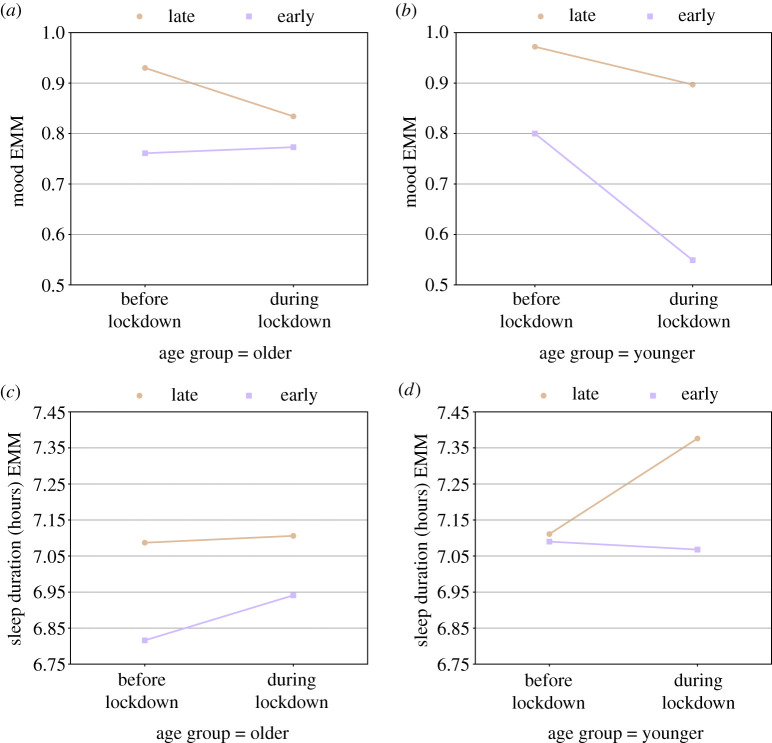
Effects of the lockdown on different combinations of age groups and chronotypes: (*a*+*b*) mood, and (*c*+*d*) sleep duration recorded by the smartwatch. The *y*-axis represents the estimated marginal means (EMM) for the examined well-being indicator.

We also found a significant interaction between lockdown, age group and chronotype in mood (*p* = 0.02) and sleep duration recorded by the smartwatch (*p* = 0.04), suggesting a different effect of the lockdown on different combinations of age group and chronotype with respect to these two indicators. Specifically, for the older age group, the decrease in mood was mainly expressed in late chronotypes (figure 5*a*), whereas for the younger age group the decrease in mood was mainly expressed in early chronotypes (figure 5*b*). Similarly, for the older age group, the increase in sleep duration recorded by the smartwatch was mainly expressed in early chronotypes (figure 5*c*), whereas for the younger age group, it was mainly expressed in late chronotypes (figure 5*d*). *Post hoc* analysis revealed that the change of mood between the two time periods was significant in the younger age group for early chronotypes (*p* < 0.01, Cohen’s *d* = 0.55). The change in sleep duration was significant or near significant in the younger age group for late chronotypes (*p* < 0.01, Cohen’s *d* = 0.31) and in the older age group for early chronotypes (*p* = 0.09, Cohen’s *d* = 0.12).

## 4. Discussion

The current study demonstrates that at the entire population level, the second lockdown in Israel resulted in lower mood, reduced resting heart rate, fewer social interactions, lower physical activity (number of steps and sport duration), increased sleep duration and later MSF and MSW. We did not find an effect on stress, sleep quality or SJL. In general, this part of our analysis is in agreement with recent studies, which were mostly based on self-reported questionnaires. These, studies, which compared control (before the pandemic) and lockdown periods found that lockdowns resulted in longer and later sleep on weekdays like we did, but also reduced SJL [5,10,12]. This difference may stem from a different, acute response to the first lockdown, and/or a lasting effect of the pandemic which did not return to the pre-pandemic situation. Regarding mood, our results are in line with those of Ingram and colleagues who found lower mood during lockdowns [39]. The increase in sleep duration was evident from both the questionnaire and the smartwatch data, and is in agreement with several recent studies [5–7,9,10,40], which found a longer sleep duration during lockdowns. It should be noted, however, that the effect sizes found in the above-mentioned cases were relatively small.

A more complex and interesting picture and larger effect sizes appear when we separately examine different subpopulations based on age, gender and chronotype. Specifically, our analyses indicate that sleep duration shows a significant increase in the late chronotypes of the younger group, and a marginally significant increase in the early chronotypes of the older group. Late chronotypes, especially in the age of our younger group which usually work or study and have younger children (also in our study), normally suffer sleep loss [24]. With no need to wake up the children or go to everyday obligations, the more relaxed social schedule induced by the lockdown could allow them to wake up later without using an alarm clock and increase their sleep duration [12]. In the older group, the opposite result was observed. An increase in sleep duration was documented in the early chronotype, and no effect was observed in the late chronotypes. It is possible that this population could not perform some early social activity like group sports, but other explanations may also be possible. Unlike previous studies [6,7,10], we did not find an effect on SJL, even when we examined subpopulations: both MSW and MSF were delayed similarly in all subpopulations. In contrast to the other studies, our control measurements were conducted between the first and second lockdowns, hence within a period of time that even without lockdown included many social changes (e.g. work from home, and distant education for some children age groups).

We found that in general the lockdown resulted in lower mood levels. This effect was evident in both age groups, but the decline was significant only in the early chronotypes of the younger group, which had higher mood levels before the lockdown and lower mood levels during the lockdown compared with older participants (figure 5*a*,*b*). A similar pattern was found in the number of steps per day, which decreased mainly in the younger participants. Such association between levels of physical activity and mood during the COVID-19 pandemic was previously reported [39]. Interestingly, this group also did not increase sleep duration, in contrast with the young late chronotypes, which may contribute to the opposite effects of the restrictions on mood. Stress levels did not change in our general study population, but interestingly that can be attributed to opposite effects in men and women: stress levels in men significantly decreased during the lockdown, but significantly increased in women (figure 4*a*). Women also experienced a more considerable decrease in the number of social encounters (figure 4*b*)—40% versus 20% in men. Physical activity and social interactions, including casual everyday meetings (e.g. with a seller at a coffee shop or a public transportation driver), are known to be correlated with mood, have beneficial effects for clinical or subclinical depression or anxiety, and as a means of upgrading life quality and well-being [41–45].

The opposite response of men and women in stress levels could result from social differences between them. In Israel, more women (21%) lost their job (fired or sent to unpaid vacation) compared to men (15%, The Israeli Ministry of Finance). Similarly, a study conducted in the US found that mothers with young children have reduced their work hours four times more than fathers [46]. Moreover, schools and daycares were closed during lockdowns and parents had to stay at home with their children. Studies in Germany reported that during the COVID-19 lockdown men were more concerned about paid work while women were more worried about childcare and shouldered more childcare work [47,48]. Furthermore, mothers experienced a greater decline in employment satisfaction while fathers’ well-being was less affected and their family satisfaction even increased [49]. Another factor that could affect stress levels is domestic violence against women, which according to Israeli Police data, increased during lockdowns. Specifically, during 1 March and 15 April there was a 16% increase in the number of cases opened and 31% increase in phone calls to the police emergency line reporting domestic violence against women, compared to the same period last year (Israel Police data).

The observed effects may have consequences beyond merely well-being. Positive mood has been associated with enhanced immune function, while negative emotions such as stress, depression, and loneliness are correlated with the suppression of the immune system [50–52]. Misalignment of daily rhythms (as a result of shift work, frequent time-zone travel and even SJL) and short sleep duration have adverse effects on our immune system function [32]. Studies showed that these factors increase infection probability [28,30,53–55] and morbidity in response to viral infection [29], and reduce vaccine protection [56,57]. Therefore, it is possible that the beneficial effects of lockdown in fighting COVID-19 results not only from the reduced social interactions (clearly observed in our study), but can also be attributed to the reduction in SJL and increase in sleep duration, which are beneficial to the immune system [56,57]. It is important to know that even one night of sleep deprivation before vaccination reduces vaccine protection [55,56].

A limitation of this study is the absence of richer sociodemographic information such as employment status and profession. Such information, if existed, could shed more light on our findings, and help identify additional effects. However, it is important to note that even if such data were available, incorporating it as factors in our analyses would most probably require a considerably larger sample size. Moreover, our main analyses were designed as mixed ANOVA tests with a dependent variable having only two repeated measures—before and during the lockdown. In other words, although data were collected for relatively long periods before the lockdown and during the lockdown, the two time periods were reduced to two averaged values. Future research should consider using more longitudinal data processing methods, such as linear mixed models or growth models, which may lead to additional insights.

Together, our and others’ results [58] suggest that young early chronotypes who did not increase their sleep duration, reduced activity level and suffered from significantly reduced mood, and women, who suffered an increase in stress levels and a greater decline in social encounters, are more adversely affected by the lockdowns. Moreover, since our results span well into the pandemic, after the participants have already gone through a first lockdown and should therefore be acquainted with it in some way, it is reasonable to assume that the effects we found are long-lasting and not only acute responses to the lockdown. These results are important by themselves, and suggest that these subpopulations, and especially early young chronotypes who usually are not in the focus of social support, should get special attention during lockdowns. Moreover, because these negative effects of the lockdowns can also suppress the immune system and result in higher infection probability and morbidity in response to viral infection and reduced vaccination efficiency, it is even more important to support the more negatively affected groups, and offer them guidance and recommendations on how to reduce these negative effects. We suggest that such knowledge can enable authorities to focus specific support actions to specific subpopulations to improve well-being, and consequently also the immune function.

The COVID-19 lockdown, together with our data collection framework, offer a unique opportunity to assess the effects of more relaxed social schedules on a multitude of daily rhythms and well-being indicators, which can be beneficial in our battle against the COVID-19 pandemic, and for the post COVID-19 ‘new normal’ life. Using the combination of data collected from wearable devices and self-reported questionnaires from the same individuals, we are able to evaluate the effects of the lockdown on different ages, chronotypes and sexes for various well-being indicators. Based on the observed effects, we can suggest some recommendations for better coping with lockdowns and enhancing our immune function. These recommendations include proper sleep timing and duration, engagement in physical activity, and proper light exposure during the day and darkness during the night. The ability to work from home during the COVID-19 pandemic and the more flexible schedule may allow us to recommend and maintain healthier daily schedules to improve well-being and health, including reduced infection chances and severity, which can be implemented during, but even more importantly after, the COVID-19 pandemic.

## Endnotes

1 MSF is considered to be a standard measure of an individual’s chronotype [36–38]. The MSF of participants before the lockdown represents sleep behaviour on a regular time period and therefore is suitable for estimating their chronotype [10].

2 It should be noted, however, that the internal distribution of ages within each age group differs between men and women. For the younger age group, very young participants prevail in men, whereas women present a more even distribution. For the older age group, the distribution is right skewed in women and somewhat evenly distributed in men.

## Data accessibility

An aggregated version of the data that allows to reproduce all the results described in the study is attached as electronic supplementary material.
